# GAPDH and PUM1: Optimal Housekeeping Genes for Quantitative Polymerase Chain Reaction-Based Analysis of Cancer Stem Cells and Epithelial-Mesenchymal Transition Gene Expression in Rectal Tumors

**DOI:** 10.7759/cureus.12020

**Published:** 2020-12-10

**Authors:** Litika Vermani, Rajeev Kumar, Nachimuthu Senthil Kumar

**Affiliations:** 1 Biotechnology, Mizoram University, Aizawl, IND; 2 Research, Cachar Cancer Hospital and Research Centre, Silchar, IND

**Keywords:** reference gene, gapdh, pum1, rectal tumor, cscs, emt

## Abstract

Background

The overwhelming majority of published articles have taken colon and rectal cancer as a single group, i.e., colorectal cancer, when normalizing gene expression data with housekeeping genes (HKG) in quantitative polymerase chain reaction (qPCR) experiments though there are published reports that suggest the differential expression pattern of genes between the colon and rectal cancer groups and hence the current experiment was attempted to find out the optimal set of housekeeping genes from the list of common HKG for rectal tumor gene expression analysis.

Methods

The expression of five potential housekeeping genes GAPDH, RPNI, PUM1, B2M, and PMM1 was analyzed through qPCR and Bestkeeper software (http://www.wzw.tum.de/gene-quantification/bestkeeper.html) in 20 stage II-IV rectal cancer samples to check for uniformity in their expression pattern. Cancer stem cell (CSC) marker ALDH1 and epithelial-mesenchymal transition marker (EMT) markers E cadherin, vimentin, Twist, and SNAI2 expression were evaluated in conjunction with the two optimal reference genes in 10 rectal cancers as part of validation.

Results

The standard deviation of the cycle threshold value of GAPDH was found the lowest at 0.65 followed by RPN1 at 0.88, PUM1 at 0.94, PMM1 at 0.94, and B2M at 1.21 when analyzed with BestKeeper software. Using GAPDH and PUM1 as the reference gene for the validation phase, rectal cancer patients with stage III/IV showed a 4.79-fold change (P=0.006) in ALDH1 expression, and an 11.76-fold change in Twist expression (P=0.003) with respect to stage II rectal tumor when normalized with GAPDH and PUM1.

Conclusion

GAPDH and PUM1 can be used as an optimal set of housekeeping genes for gene expression-related experiments in rectal tumors. ALDH1 and Twist were found significantly overexpressed in stage III/IV rectal tumors in comparison to stage II rectal cancer. Genes associated with cancer stem cells and EMT markers could be optimally analyzed by normalizing them with GAPDH and PUM1 as housekeeping genes.

## Introduction

Colorectal cancer is the third most common cancer in the world and the third leading cause of cancer death in men and women in the United States, whereas it is the fourth most common cancer in men and the third most common cancer in women in India, where rectal tumor constitutes nearly 39% and colon cancer 61% of the cancer cases [1-2] with a 2.6 crude rate for both colon and rectum cancer development [3]. Colorectal cancer is heterogeneous, i.e. it varies both genotypically and phenotypically [4].

In the majority of studies where colon and rectal cancer are studied as a single unit, i.e. colorectal cancer, the effort to look deep into their biology to generate deeper insight into their pathophysiology are sometimes masked. Studying the expression pattern of genes in rectal cancer can provide novel insights into our understanding of this disease. Differential gene expression is the most common and widely used application of real-time quantitative polymerase chain reaction (qPCR) in today’s molecular health research. Reverse transcriptase quantitative PCR (RT-qPCR) is the most sensitive, precise, powerful, and robust technique available for the analysis of gene expression in a wide variety of applications ranging from basic and applied research to molecular diagnostics in cancer [5]. To accurately quantitate mRNA levels in different types of study samples, it is critical to normalize the RT-qPCR data with a gene that is known to express stably in all experimental conditions to compensate for the difference in the initial amount of input mRNA used as a template during cDNA synthesis, uneven subjective pipetting, reverse transcription yield, PCR efficiency and changes in the experimental condition [6]. The normalization of the mRNA levels between different samples can be achieved by using the set of optimal reference genes [7].

Widely used reference genes such as glyceraldehyde 3-phosphate dehydrogenase (GAPDH), glucuronidase beta (GUSB), β-actin, and β-globin were commonly and randomly used in an overwhelming number of published reports without their validation on the sample type, and they were reported as not stably expressed [8]. This approach has created flawed results that may not be reproducible and create a pseudo sense of knowledge gain to our understanding of the biological problem that we intend to address. Also, minimum information for publication of quantitative real-time PCR experiments (MIQE) guidelines recommends validating reference genes before using them for a tumor type and using more than one reference gene during normalization of the target gene expression value across multiple test samples, though in practice, it is rarely universally followed. Therefore, the aim of this study is to identify and validate suitable HKG for the evaluation of gene expression in rectal cancer. Five promising reference genes GAPDH, Pumilio RNA binding family member 1 (PUM1), beta-2-microglobulin (B2M), ribophorin I (RPN1), and phosphomannomutase 1 (PMM1) were selected from different publications [7,9-10] to assess their expression stability in rectal tumors as part of the screening phase. The expression of cancer stem cells (CSCs) and epithelial-mesenchymal transition (EMT) molecular markers have been shown to impact the clinical outcome in a wide variety of solid malignancies including rectal tumors [11] and could be the reason for their aggressive tumor biology and metastasis. CSCs marker aldehyde dehydrogenase 1 (ALDH1) and EMT markers E cadherin, vimentin, and Twist were used to validate the set of reference genes from the screening phase. To our best of knowledge, this is the first report that has analyzed the most stably expressed reference genes among GAPDH, RPNI, PUM1, B2M, and PMM1 in rectal tumors.

## Materials and methods

Study design

The current study was divided into the screening and validation phases. For the screening phase, five potential reference genes, GAPDH, RPNI, PUM1, B2M, and PMM1, were selected from the available literature and subjected to evaluation on 20 rectal tumor samples in triplicates. The same sets of potential housekeeping genes were assessed on six normal rectal tissues. Post analysis, two suitable HKGs were identified that showed stable expression and were subsequently used in the validation phase on 10 new rectal cancer tissue samples. In the validation phase, the selected HKG was used in the normalization of ALDH1, E cadherin, vimentin, and Twist in rectal samples to assess their performance.

Materials

Treatment-naïve rectal cancer tissue samples from the year 2015 to 2019 stored in RNA-later preservative media at -20ºC were used in this study. Pathologically confirmed rectal cancer tumors ranging from stages II to IV and with tumor content ranges from 50%-80% were only taken for the study. Samples with normal rectal pathology were selected for control. This study was approved by the institutional review board (IRB).

Reference gene selection

A thorough literature survey was done to find out genes having uniform expression across colorectal/colon/rectal tumors [7,9-10]. We found GAPDH, RPNI, PUM1, B2M, and PMM1 as promising reference genes. Their primers were designed based on exon-exon junctions using the National Center for Biotechnology Information (NCBI) tool Primer-BLAST. Some of the primers were also designed with the help of the Integrated DNA Technologies website. The list of primers is given in Table 1.

**Table 1:** List of primers of the reference genes used in the current experiment

Genes	Sequence	Exon-exon junction	Source
GAPDH	F TGCACCACCAACTGCTTAGC R GGCATGGACTGTGGTCATGAG	Yes	Published [12]
PUM1	F TGAGTTTATTCCTTCAGACCAGCAG R GCAAATACCTGTCCCTTAAACGCA	Yes	Self-designed
B2M	F GCCCAAGATAGTTAAGTGGGATCG R CCTCTAAGTTGCCAGCCCTCC	Yes	Self-designed
RPN1	F TGGACTTAAAGCACACAGGAG R CAGCAGGAAGGATGGTCTTAAA	Yes	Self-designed
PMM1	F GGCTCGCCAGAAAATTGACCC R TACTGCACCGTCCCGTTCTC	Yes	Self-designed
ALDH1	F TGTTAGCTGATGCCGACTTG R TTCTTAGCCCGCTCAACACT	Yes	Published [13]
Vimentin	F TCTCTGAGGCTGCCAACCG R CGAAGGTGACGAGCCATTTCC	Yes	Published [14]
Twist	F GCCGGAGACCTAGATGTCATT R TTTTAAAAGTGCGCCCCACG	Yes	Self-designed
E cadherin	F CCACCACGTACAAGGGTCAG R TGCCATCGTTGTTCACTGGA	Yes	Self-designed

RNA extraction

Pathologically confirmed rectal cancer tissue samples stored in RNA-later were used for the extraction of RNA using the AllPrep DNA/RNA kit (Qiagen, Germany). Twenty to 25 mg of tissue was cut with the help of sterile scissors and was homogenized in the RLT Plus buffer (Qiagen, Germany) with Takara Biomasher (Takara Bio Inc., Shiga, Japan) for 20-30 seconds until the tissue was completely homogenized. The homogenized lysate was transferred in a DNA spin column, centrifuged, and 70% ethanol was added to the flow-through. This flow-through, after thorough mixing, was transferred to an RNA spin column, and post-centrifugation, the flow-through was kept in a new 1.5 ml centrifuge tube at -80 °C for future protein precipitation. On-column DNase treatment was done to remove any DNA contamination from the RNA column. This was followed by washing steps and, finally, the RNA was eluted in 50 µl of nuclease-free water and stored at -80 °C.

The quality of RNA was checked by agarose gel electrophoresis and the quantity was measured using Qubit Fluorometer (Thermo Fisher Scientific, Waltham, Massachusetts).

cDNA synthesis

One μg of good quality RNA was taken as the template for converting it into cDNA using the Quantitect reverse transcription kit (Qiagen, Germany) according to the instructions by the manufacturer. The RNA samples were treated with genomic DNA elimination buffer so as to remove any trace amount of gDNA from the RNA sample. This RNA was converted into cDNA by adding the Quantiscript reverse transcriptase enzyme, Reverse transcription (RT) primer mix, and Quantiscript RT buffer 5X, followed by PCR reaction and no RT control with the following cycle condition:

Genomic DNA elimination: 42°C at two minutes followed by keeping it on ice.

cDNA synthesis: 42°C for 15 minutes followed by 95°C for three minutes and immediately kept on ice to stop the reaction.

qPCR: Sybr green chemistry was used in the Quantitect Sybr Green Master Mix (Qiagen, Germany). Specific primers were screened using cDNA/gDNA/NTC as the template in triplicates and melt curve analysis for ascertaining their specificity. The qPCR condition was set up as per the following:

Hold 95°C for five minutes

95°C for 10 seconds

62°C for 30 seconds - acquire to green channel. 40 cycles

Melt: 50°C to 99°C with 1°C interval

Gain optimization for green channel: 1 to 3

Sample maximization assay for reference gene expression analysis in triplicates was performed. The average cycle threshold (Ct) value of the HKG was analyzed using Bestkeeper to select the optimal reference gene that showed uniform expression through all the rectal tumor samples and had a standard deviation of less than 1.

Determining the stability of housekeeping genes through an Excel-based tool using pair-wise correlation (software: Bestkeeper version 1; http://www.wzw.tum.de/gene-quantification/bestkeeper.html).

The average Ct values of all the samples tested with different housekeeping genes were tabulated in Bestkeeper software version 1 [15]. The Bestkeeper software helps determine the best-suited standards that can be used as housekeeping genes for our experimental setup. This software determines the control reference genes on the basis of standard deviation (SD) [15]. Those genes that showed the lowest SD value in the Bestkeeper software are the most stable expressed reference gene in terms of gene expression and vice versa [16].

Statistical analysis

Chi-square with Fisher’s exact test was used for analyzing the correlation. GeneGlobe online software (Qiagen Incorporation, Germany) was used to analyze the CSCs and EMT gene expression data in the validation phase, which was normalized with the geometric mean of the reference genes and to calculate fold change with P-value. In all tests, a two-tailed P-value was considered and the test was considered statistically significant when P ≤ 0.01.

## Results

Five potential housekeeping genes, GAPDH, RPNI, PUM1, B2M, and PMM1, were assessed for their expression stability on qPCR using Sybr Green Chemistry and melt curve analysis. All of these genes were analyzed for their stability in 20 rectal tumors and six normal rectal samples through qPCR as part of the screening phase through Bestkeeper software. The crossing point (CP) value in rectal cancer samples for GAPDH ranges from 13.69 to 17.18, CP value for PUM1 ranges from 18.37 to 21.52, CP value for B2M ranges from 9.72 to 14.53, CP value for RPN1 ranges from 16.66 to 19.64, and CP value for PMM1 ranges from 19.89 to 23.71 as represented in Table 2. Whereas in normal rectal samples, the CP value for GAPDH ranges from 15.35 to 17.20, PUM1 18.40 to 20.59, B2M 10.42 to 11.99, RPN1 16.66 to 18.65, and PMM1 20.35 to 21.64.

**Table 2:** Bestkeeper software output of five housekeeping genes tested in rectal cancer

	GAPDH	PUM1	B2M	RPN1	PMM1
n	20	20	20	20	20
geo Mean [CP]	15.53	19.70	12.13	18.11	21.84
ar Mean [CP]	15.55	19.73	12.22	18.14	21.87
min [CP]	13.69	18.37	9.72	16.66	19.89
max [CP]	17.18	21.52	14.53	19.64	23.71
std dev [± CP]	0.65	0.94	1.21	0.88	0.94
CV [% CP]	4.18	4.74	9.92	4.85	4.29

The CP or cycle threshold was calculated from the amplification plot of the individual reaction graph post the application of 0.07 thresholds. The geometric mean for GAPDH, RPNI, PUM1, B2M, and PMM1 were reported as 15.55, 19.73, 12.22, 18.44, and 21.87 in rectal tumor samples, respectively. Table 2 showed the standard deviation of GAPDH that was reported the lowest at 0.65 followed by RPN1 at 0.88, PUM1 at 0.94, PMM1 at 0.94, and B2M at 1.21 in rectal tumors.

As part of the validation phase, Table 3 showed CSCs and EMT gene expression validation using GAPDH and PUM1 as a combination of HKGs.

**Table 3:** CSCs and EMT gene expression validation using GAPDH and PUM1 as reference genes CSCs: cancer stem cells; EMT: epithelial-mesenchymal transition

Sl. No	Gene	Fold regulation in stage III/IV rectal tumor with respect to stage II rectal tumor		Fold regulation in metastatic rectal tumor with respect to stage II rectal tumor	
	Gene	Fold change	P-value	Fold change	P-value
1	ALDH	4.79	0.006	7.14	0.075
2	E cadherin	1.70	0.005	-1.22	0.244
3	Vimentin	4.84	0.024	2.07	0.537
4	Twist	11.76	0.003	5.05	0.015

In the validation phase, 10 new rectal tumors were evaluated taking the geometric mean of GAPDH and PUM1 as the reference gene. In the validation phase, rectal cancer patients with stage III/IV showed a 4.79-fold change (P=0.006) in ALDH1 expression, 1.70-fold change in E cadherin, 4.84-fold change in vimentin (P=0.02), and 11.76-fold change in Twist expression (P=0.003) with respect to stage II rectal tumor when normalized using GAPDH and PUM1 reference genes. Similarly, when metastatic rectal cancer cases were compared with stage II rectal tumors, a 7.14-fold difference in ALDH1, -1.22-fold change in E cadherin, 2.07-fold change in vimentin, and 5.05-fold change in Twist (P=0.015) were reported.

## Discussion

The majority of published literature has considered colon and rectal cancers in a single colorectal cancer type when normalizing gene expression data with reference genes in qPCR experiments, even though there are reports that suggest a difference in the expression pattern of genes between colon and rectal cancer groups [17]. In the current experiment, we attempted to ascertain the optimal set of reference genes for gene expression analysis in rectal tumor and rectal normal samples.

The genes evaluated for rectal cancer and normal rectal samples in our study included GAPDH, RPNI, PUM1, B2M, and PMM1, which are involved in the process of transcription/translation; other genes are involved in nucleotide metabolism, are a part of the cytoskeleton, etc. [18-19]. The results suggested that the expression of the GAPDH, PMM1, and PUM1 genes was most stable in malignant colon samples with less than 10% variability that correlates with our findings. The genes that showed the highest variability (more than two-fold) in colorectal cancer included HPRT and ADA [6].

The Cancer Genome Atlas reported no clear distinction of mRNA and miRNA expression levels between non-hypermethylated colon cancer and rectal tumors; however, some differences were found in gene expression patterns between the right colon and rectum [20]. However, several reports suggested a difference in the expression pattern of genes in colon and rectal tumors [17,21-22] along with the significant differences in the mutation level of APC and KRAS mutation between colon and rectal cancer [23]. This indicates a mixed report on whether there exists a difference in the expression pattern of colon and rectal cancer. Though to make sure the correctness of the inference from gene expression studies based on qPCR in either rectal or colon tumors, independent validation of HKG makes sense in each site. Since the objective of our study was to find optimal sets of HKG only in rectal tumors; therefore, we evaluated the five potential HKG in only rectal cancer and normal rectal samples.

The expression of 13 endogenous control genes was evaluated in primary colorectal cancer tumors, as well as their adjacent normal tissues by Kheirelseid EA et al., to look for the most suitable. The genes evaluated in their study included hypoxanthine-guanine phosphoribosyltransferase 1 (HPRT), beta-actin (ACTB), mitochondrial ribosomal protein L19 (MRPL19), beta-2-microglobulin (B2M), GAPDH, and PPIA (peptidyl-prolyl isomerize A), where they reported B2M and PPIA as the most stably expressed genes on colorectal tumor [24]. However, in our study, we found B2M as the least stably expressed gene in rectal cancer among the studied group of potential reference genes.

GAPDH, cyclophilin, and B-actin are the most common reference gene used for gene expression studies but recent studies have suggested that there is high variability in their expression as a result of hypoxia in tumor tissues [25]. According to Bestkeeper software version 1, the most stable and suitable housekeeping genes across our colorectal cancer samples are GAPDH 2 (HGK 1) with standard deviation 0.71 and p = 0.01, PUM1 2 (HGK 2), and RPN1 (HGK 3) are equally good and have the same standard deviations 0.94 and p=0.001. PMM1 2 (HGK 5) could also be used as its standard deviation was 0.94 with p = 0.001 but B2M (HGK 4) was not found to have a stable expression in our set of samples as the standard deviation was more than 1. However, in rectal tumors, we found GAPDH, PUM1, and RPN1 with the most stably expressed reference genes.

The geometric mean of GAPDH and PUM1 when used to normalize the expression of CSCs and EMT genes during the validation phase, tumor, nodes, and metastases (TNM) stage III/IV rectal cancer patients showed a 4.79-fold change (P=0.0068) in ALDH1 expression, 1.70-fold change in E cadherin, 4.84-fold change in vimentin (P=0.02), and 11.76-fold change in Twist expression (P=0.003) when compared with stage II rectal tumor when normalized using GAPDH and PUM1 as reference genes. A similar finding was also reported by Zhou et al. [26] and Yong et al. [27]. Taking geometric mean help in controlling the outlier values and using more HKG than 1 increases the accuracy of qPCR assay measurement [12]. The current study has some limitations in terms of having a limited number of samples for its validation study. Future studies with more number of rectal cancer samples will add more insights and strength to this field of study.

## Conclusions

Using BestKeeper software, GAPDH, PUM1, and RPN1 were identified as the most suitable reference gene among 20 rectal tumor cases irrespective of their stage II-IV, pathology, and treatment regime, whereas B2M showed the least stable expression among the studied potential reference genes for rectal tumor. The GAPDH and PUM1 combination was used successfully for normalization in the experiment related to the CSCs and EMT markers ALDH1, E cadherin, vimentin, and Twist expression in rectal tumors. In stage III/IV rectal tumor, ALDH1, and Twist were found significantly overexpressed in comparison to stage II rectal cancer. GAPDH, RPN1, and PUM1 could be used as a suitable reference gene for gene expression-based qPCR experiments in rectal tumors.
